# The healthy behaviours and COVID-19 mortality among Iranian women: a case–control study

**DOI:** 10.1186/s12905-021-01512-0

**Published:** 2021-10-17

**Authors:** Farhad Pourfarzi, Shima Rahim Pouran, Abdollah Dargahi, Chiman Karami, Nasrin Fouladi, Hamed Zandian, Telma Zahirian Moghadam

**Affiliations:** 1 Digestive Disease Research Center, Ardabil University of Medical Sciences, Ardabil, Iran; 2 Social Determinants of Health Research Center, Ardabil University of Medical Sciences, Ardabil, Iran; 3 School of Medicine and Allied Medical Sciences, Ardabil University of Medical Sciences, Ardabil, Iran; 4 Department of Community Medicine and Family, Ardabil University of Medical Sciences, Ardabil, Iran

**Keywords:** Woman health, COVID-19, Mortality, Health protocols, Behaviours

## Abstract

**Background:**

Women are among the susceptible groups to Coronavirus disease-19 (COVID-19) in Ardabil, north-west of Iran, despite the current global status. The underlying causes of high incidence and fatality rate of women in Ardabil are not fully understood. Hence, this study aimed to investigate the healthy behaviours in women of Ardabil and its relationship with COVID-19 mortality.

**Methods:**

We conducted a case–control study to compare the adherence to health protocols and behaviours with respect to COVID-19 between the infected (261 patients) and healthy (515 persons) women. Health protocols and behaviours such as using mask, gloves, disinfectants, history of travelling and contacting, and attending various gatherings and places during the COVID-19 pandemic along with demographic variables were defined as independent variables, and COVID-19 death rate was defined as the dependent variable. Multivariable logistic regression methods were used to explore the risk factors associated with COVID-19 mortality.

**Results:**

Chi-square and Fisher tests showed significant differences between infected and healthy women in terms of history of contact and traveling (*p* < 0.05), wearing mask (*p* < 0.001), going to work place (*p* < 0.001), and attend public gatherings (*p* = 0.038). Multivariable logistic regression disclosed that the age group over 80 years: 8.97 times (95% CI 2.27–29.85), women with underlying chronic diseases: 4.14 times (95% CI 1.61–10.64), and obese women: 3.01 times (95% CI 1.04–6.03) were more likely to die from COVID-19 than other women.

**Conclusion:**

Considering the high incidence and mortality rate in Ardabil women due to COVID-19 and the corresponding health behavioural factors, special emphasis should be given to the increase of women awareness on the importance of healthy behaviours, diet, and life-style.

## Introduction

Since late 2019, the new human coronavirus, Coronavirus disease 2019 (COVID-19), was initially reported in Wuhan, China, and spread rapidly all over the world, and has now become a pandemic and an international health concern. It has had many human, economic, and social consequences [1]. Globally, the number of people infected with COVID-19 has reached to more than 178 million out of which more than 3.8 million were died due to this outbreak untile June 19, 2021. From the reports, twelve countries included United States, India, Brazil, France, Turkey, Russia, Britain, Italy, Argentina, Colombia, Spain, and, Germany bear more than 3.5 million confirmed cases of COVID-19 [2]. In Iran, out of 3.03 million individuals infected by COVID-19, 82 thousand people lost their lives [3].

The reports on the COVID-19 infected groups in most countries, including Iran showed that twice as many men as women are susceptible to COVID-19 [4]. The rate of mortality of men in New York was twice as that of women while it was 70% and 63% higher in China and Europe, respectively [5]. In Iran, based on the national statistics on the patients admitted to COVID-19 hospitals, 63% were men while women constituted 37% [6].

Although, all people of the community, from young to old and men and women, are at risk of COVID-19, age, and sex are two critical factors influencing the incidence or severity of the symptoms. The incidence and severity of COVID-19 in men is affected by a number of factors, as identified via various global studies, including biological factors e.g., genetic, hormonal, and enzymatic factors, plus social profile, job, and additional economic responsibility of men. These factors together with cultural perspectives such as misconceptions of men for being superior and more robust than women, forge them to pay little attention to their health, fail to care for attending medical centres, doing test, and comply with hygiene principles of frequent hand washing, wearing masks and gloves [7–11]. Collectively, these factors put men at higher risk of infection and fatality due to COVID-19.

On the other hand, chronic diseases such as cardiovascular disease (high blood pressure, diabetes, coronary heart disease, and cerebrovascular disease) are also more common in men than women, resulted from the unhealthy behaviours such as inactivity, unhealthy diet, and smoking which are comparatively prevail in men [12].

In most parts of the world, men are more likely than women to develop Covid-19 and experience a more severe illness, so most studies have focused on men to identify the most important contributors to Covid-19 mortality and morbidity. On the other hand, the incidence rate of Covid-19 in Ardabil, as one of the provinces of Iran, unlike other parts of the world, was higher in women than men. On the other hand, according to the COVID-19 dashboard in Iran, more than 63% of Covid-19 patients in Ardabil province are women. Considering that there is no known reason for the higher incidence of Covid-19 in men or women around the world, as Covid-19 is an unknown virus with unpredictable effects and may change the incidence rates of Covid-19 by sex in the future in other parts of the world. Therefore, this study is designed to identify the most important risk factors for COVID-19 among women with Covid-19 and healthy women to ultimately provide policies specific to women to prevent and control Covid-19. Considering the contradiction between the Ardabil province and most parts of the world in higher prevalence of COVID-19 among women than men, this study aimed to investigate the healthy behaviours in women of Ardabil and its relationship with COVID-19 mortality.

## Methods and materials

### Study area

This study was conducted in Ardabil province, one of the largest provinces in the north-west of Iran. According to the Statistical Centre of Iran, the total of Ardabil province inhabitants was estimated to be 1.3 million in 2019, wherein 48% were women. Administratively the province is divided into 10 cities, and in total, there are 13 public hospitals and 5 private hospitals in the province.

### Study design

The study design was an unmatched case–control study. The source populations for this study were women who were living in Ardabil, Iran.

### Study population

The cases were women hospitalised for Covid-19 in public and private hospitals designated as Covid-19 treatment centres from April 2 to October 22 2020. The controls were healthy women who were not infected with Covid-19 at all. We included women who autonomously signed the informed consent or had expressed their consent in the medical record to use their data in various studies. Sampling was performed from May 19 to October 22, 2020.

### Sample size

The sample size was determined using a formula for two population proportions and was calculated using G-Power software version 3.1.9.7 statistical software. The prevalence of Covid-19 among women in Ardabil is 63%. The expected ratio in the control group (expected proportion in controls) equal to 0.05, the confidence level of 0.95, the study power 0.8, and the odds ratio of exposure equal to 2.5. Finally, the case group's sample size was chosen as 261 people to examine exposure's behavioural aspects. The minimum control group's sample size was calculated to be about 515 people, where the ratio of the control group's sample size to the case group was considered twice.

$$N = \frac{{4\left( {z_{{1 - \frac{\alpha }{2}}} + z_{1 - \beta } } \right)^{2} }}{{\left( {\ln \hbox{OR}} \right)^{2} \pi \left( {1 - \pi } \right)}} \cong 261$$

Incomplete questionnaires were replaced randomly with other questionnaires in both case and control groups. Finally, 261 and 515 questionnaires were collected for cases and controls, respectively. The total sample size (cases and controls) was 776 people.

## Sampling technique

### Cases

Eight hospitals (7 public and one private) among the hospitals located in Ardabil province (13 public hospitals and 5 private hospitals), known as Covid-19 patients admitting hospitals, were selected as the case selection source. Data related to women with COVID-19 (cases) were collected from a retrospective medical record that was recorded in the integrated information system of COVID-19 patients in Ardabil province. Of the total number of patients with Covid-19 registered from these hospitals in the comprehensive patient information system, 63% were women. A random sampling method was used for selecting women with Covid-19 based on the national number.

### Controls

The control group consisted of women who had not been infected with Covid-19 since the Ardabil outbreak and had no recorded information about receiving inpatient services for Covid-19 or other infectious diseases in the last year. The data related to the control group were selected from the information registered in the comprehensive health care system of Ardabil province (Sib system) based on the national code. Sib is a electronic health system which use in Irans health system to monitor population health by healthcare centers. Women were those who did not have a Covid-19 in their family. A random sampling method was used to select healthy women.

### Eligibility criteria

Inclusion criteria for the case group included informed consent, being a resident of Ardabil and having information registered in the comprehensive system of Covid-19 patients. Female patients who were hospitalised or died for reasons other than Covid-19 were excluded from the study. For the control group, having an electronic file in the comprehensive health system and conscious consent to participate in the study are known as inclusion criteria.

### Data collection tools and methods

The present study's data collection tool was a questionnaire designed electronically and used as an information system to identify patients' health behaviours and healthy individuals. The case and control groups' health behaviours included three sections: contact and travel history during Covid-19 pandemic (last 14 days), observance of health protocols (using a mask, washing hands, using disinfectants, using gloves, history of traviling to high-risk areas and attend gatherings), and presence in different circles and places during the last 14 days. Demographic variables included age (less than 10 years to over 80 years), place of residence (provincial capital or affiliated cities), the underlying chronic disease including diabetes (type 1 & 2), cardiovascular disease, lung diseases (COPD), metabolic diseases, obesity, smoking (yes/no), and body mass index (from underweight to obese) were collected for all participants. In this study, having the underlying chronic disease was determined based on the participants' self-declaration and medical history request.

Due to Covid-19 infection as first and ding due to Covid-19 infection, hospitalisation was defined as the main outcome of this study. The death due to Covid-19 (yes/no) was considered as the dependent variable in the present study to determine its correlation with independent variables just in hospitalised women.

### Data analysis

Accurate Chi-square and Fisher tests were used to evaluate and compare the case and control groups in terms of demographic and contextual variables. Chi-square test with a significance level of 0.05 was used to compare both groups in terms of health behaviours. Our dependent variable was dichotomous (death by Covid-19, Yes/No), so multivariate logistic regression was used to investigate the relationship between independent and contextual variables with patients' Covid-19 mortality.

## Results

The case and control groups' results in terms of demographic variables and underlying chronic diseases are presented in Table 1. According to the results, the mean age of the case group was 54.57 ± 21.60 years, and the mean age of the control group was 43.80 ± 13.97 years. The groups showed a significant difference in mean age. As per results, 64% of women with COVID-19 had an underlying chronic disease from which the most common diseases belonged to the patients with cardiovascular disease. The lowest frequency in both groups belonged to the patients with metabolic diseases. There was a significant difference between the two groups in terms of distribution and prevalence of underlying chronic diseases (*p* < 0.05). The patients' overall smoking rate and healthy individuals were about one percent, and the studied groups did not differ significantly in this regard. Pursuant to the results, the prevalence of obesity and being overweight in the case group was significantly higher than that of the control group.

**Table 1:** Demographic characteristics of study participants (n = 776)

	Case (n = 261)		Control (n = 515)		Total	
	Frequency	%	Frequency	%	chi^2^	*p* value
*Age categories*						
< 10	17	6.5	2	0.4	228.24	< 0.001
10–19	3	1.1	21	4.1		
20–29	9	3.4	53	10.4		
30–39	20	7.7	119	23.0		
40–49	23	8.8	148	28.6		
50–59	44	16.9	97	19.0		
60–69	58	22.2	54	10.6		
70–79	57	21.8	20	3.9		
> 80	30	11.5	2	0.4		
*NCDs*						
Chronic disease	167	64.0	41	7.9	278.2	< 0.001
Diabetes	94	36.01	65	12.6	58.2	< 0.001
CVDs	120	46.1	32	6.2	174.4	< 0.001
Obese	106	40.6	24	4.6	161.2	< 0.001
Metabolic disease	7	2.7	2	0.4	7.99	0.005
Lung disease	25	9.6	26	5.0	5.86	0.015
*Smoking*						
Yes	2	0.8	5	1.0	0.078	0.715
No	259	99.2	512	99.0		
*BMI categorized*						
Underweight	17	6.5	14	2.7	13.28	0.004
Normal weight	42	16.1	128	24.8		
Overweight	117	44.8	227	44.0		
Obesity	85	32.6	147	28.5		

n, number; NCDs, non-communicable diseases; CVDs, cardiovascular diseases; BMI, Body Mass Index

The status of compliance with health behaviours and protocols in the two groups is provided in Table 2. Regarding communication and travel history, the results showed that the group of women with COVID-19 had a significant (8.8%) history of especially travelling to high-risk areas (2.3%), contact with the medical staff (2.7%). The 12.6% of patients with COVID-19 contacted familiar patients, wherein it was null in the case of healthy women (*p* < 0.05). In accordance with the results, women with COVID-19 used significantly less masks and gloves than control group (49.4% vs. 65.4%) and (30.7% vs. 38.9%) during the last 14 days, respectively. The results did not show a significant difference between the two groups in terms of traffic history to different places, including hospitals and health centres during the COVID-19 period.

**Table 2:** Health behaviors and protocols in among women with and without COVID-19 (n = 776)

	Case (n = 261)		Control (n = 515)		Total	
	n	%	n	%	chi^2^	*p* value
*Contact to/traveling*						
Traveling out of residence place	23	8.8	22	4.3	45.61	< 0.001
Contact to patient before illness	31	11.9	46	8.9	3.21	0.201
Polluted state travel	6	2.3	0	0	11.97	0.001
Contact to medical person	7	2.7	2	0.41	7.990	0.005
Contact to familiar Patient	33	12.6	0	0	68.26	< 0.001
Contact to unknown resource	180	69.0	1	0.2	459.4	< 0.001
*Wear …. in last 14 days*						
Mask	129	49.4	338	65.4	18.39	< 0.001
Gloves	80	30.7	201	38.9	5.08	0.024
Disinfectant	134	51.3	232	44.9	2.911	0.088
*Go to …. during COVID-19 pandemic*						
Clinic	21	8.0	43	8.3	0.017	0.897
Hospital	25	9.6	34	6.6	2.230	0.135
Health center	19	7.3	50	9.7	1.227	0.268
University	1	0.4	9	1.7	70.67	< 0.001
Work place	2	0.9	126	24.5	127.9	< 0.001
Public/private gathering	49	18.6	48	9.3	17.042	0.038

**Fig. 1:**
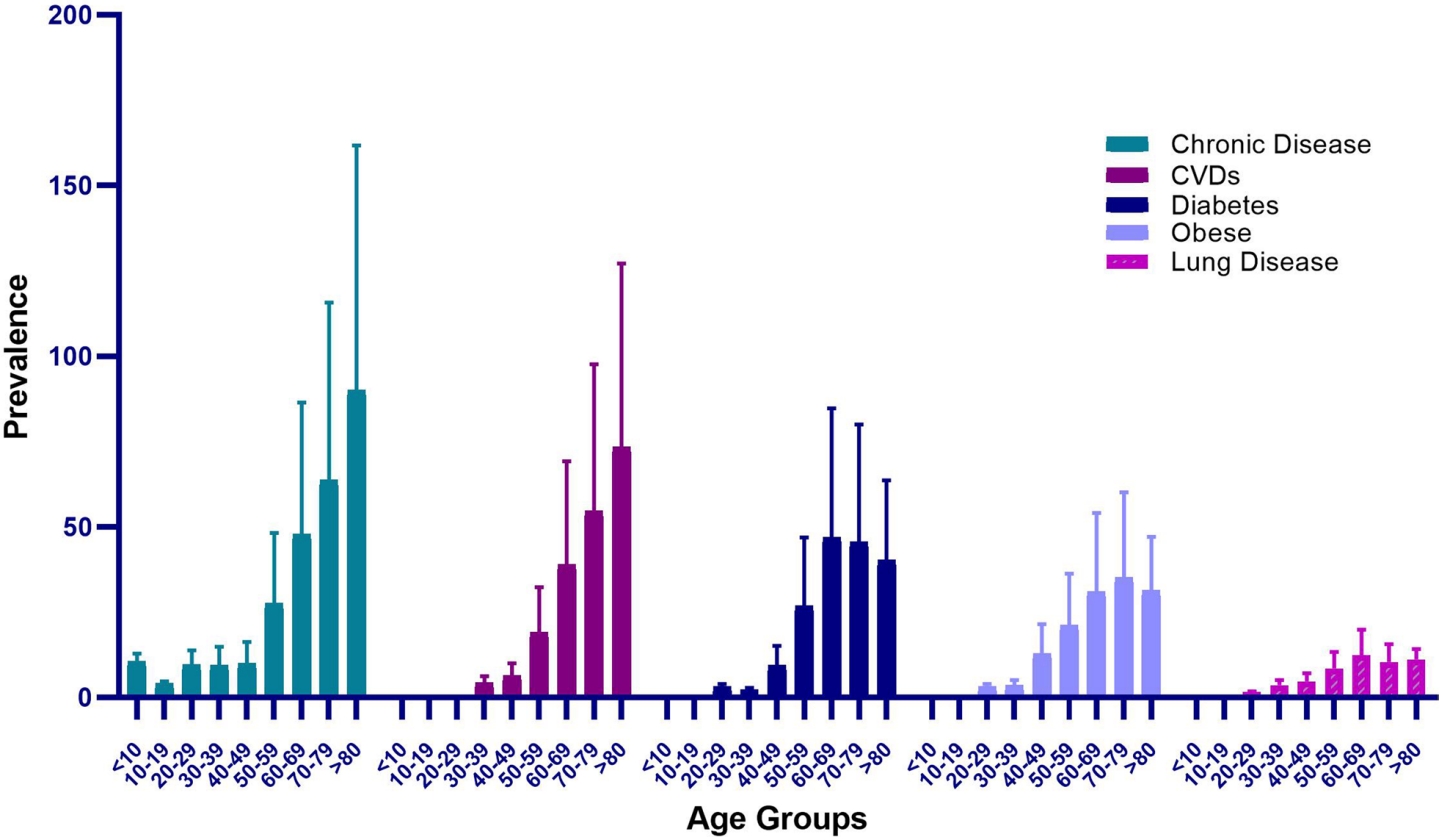
Mortality rate of women with chronic disease, cardiovascular diseases (CVDs), diabetes, obese, and lung disease due to COVID-19 with respect to the age groups

One of the main studied factors was the rate of mortality due to COVID-19 in reference to age and underlying chronic diseases (Fig. 1). From the results, the highest mortality occurred in individuals with underlying chronic diseases in the age group over 80 years. Moreover, from the women with coronary heart disease who died of COVID-19, the highest frequency belonged to the age group over 80 years. However, the women with diabetes, obesity, and underlying chronic diseases who died with COVID-19 were in the age group of 50–80 years. Significant differences were found between the age groups in terms of mortality due to underlying chronic diseases.

Based on the results provided in Table 3, the mortality rate was significantly increased with age among the women affected by Covid-19. The mortality rate of women above 80 years old was 8.97 times (95% CI 2.27–29.85) higher than that of women under the age of 30. In the case of the women with underlying chronic diseases, the mortality rate was 4.14 times (95% CI 1.61–10.64) more than that of women without chronic diseases. This also applies for the diabetes (1.44, 95% CI 1.61–10.64), cardiovascular disease (1.82, 95% CI 1.09–2.89), and obesity (2.95, 95% CI 1.26–6.89). By reason of low smoking rate and no fatality among the smoker female, this variable was eliminated from the model. Besides, there was no report of mortality in women under 30 years old group, which accordingly was eliminated from the model. As per results, the increase in body mass index, boosted the mortality rate in women with COVID-19 wherein fatality in obese women with COVID-19 was 3.01 times (95% CI 1.04–6.03) more than the died women with normal weight.

**Table 3:** Multivariable logistic regression model predicting death by population characteristic in inpatients women (n = 261)

	Odd ratio	[95% CI]	*P* value
*Age categories*			
< 30	1.00 (referent)	–	–
30–39	1.98	0.29–13.36	0.480
40–49	3.21	0.48–21.28	0.227
50–59	4.63	0.90–23.86	0.066
60–69	6.55	1.32–32.36	0.021
70–79	6.60	1.32–32.97	0.020
> 80	8.97	2.27–29.85	0.004
*NCDs*			
Chronic disease	4.14	1.61–10.64	0.003
Diabetes	1.44	1.06–3.09	0.038
CVDs	1.82	1.09–2.89	0.048
Obese	2.95	1.26–6.89	0.012
Metabolic disease	1.69	0.16–17.60	0.657
Lung disease	1.00	0.36–2.75	0.996
*Smoking*			
Yes	–	–	–
No	1.00 (referent)	–	–
*BMI categorized*			
Underweight	–	–	–
Normal weight	1.00 (referent)	–	–
Overweight	2.56	1.69–6.66	< 0.001
Obesity	3.01	1.04–6.03	0.004

CI, confidence interval; n, number; NCDs, non-communicable diseases; CVDs, cardiovascular diseases; BMI, Body Mass Index

## Discussion

The universal information on gender-related COVID-19 case fatality is rather controversial, and there is no general trend in different regions. Whilst, most countries reported higher fatality rates for male than female [13], the female to male case fatality rate is also higher in some instances [14]. Rozenburg etal. Showed that men are almost 60% more likely to be seriously sick or to kick the bucket from the complications of Covid-19 than are women [15]. Against the backdrop of global data, men are biologically and behaviourally at higher risk of sickness and death caused by COVID-19 [16]. In spite of the fact that within the widespread men die more regularly than women from Covid-19, it isn't clear whether typically due to biological differences between men and women, differences in behavioral habits, or differences within the rates of co-morbidities [15]. Nonetheless, prevalence of COVID-19 among women in Ardabil province was higher than men and the case illness and fatality were respectively 7.2% and 2.6% more than men (end of December 2020).

Large number of studies have been conducted all over the world to determine various factors, which increase the risk of infection and mortality by COVID-19. Taking into account that some factors are universally supported, higher COVID-19 infection and fatality rate of women in Ardabil province, against the global norm, brought about the involvement of some other factors. This study provided the first comprehensive assessment of the effect of healthy behaviours (contact and travel history, observance of health protocols, and presence in different circles and places during 14 days) and demographic factors (age, place of residence, underlying chronic disease (diabetes, cardiovascular, lung diseases, metabolic diseases), and obesity, and smoking) on the rate of COVID-19 case illness and fatality of female in Ardabil province. As per results of this study, significant differences were found between the healthy and infected women in terms of healthy behaviours such as the history of contact and traveling, wearing mask, going to work place, and attend public gatherings, in connection with the surveyed demographic factors, except for smoking habit. Pope etal. found that among pregnant women, some pandemic-related behaviours (avoiding public places and wearing masks) were higher than other population groups during Covid-19 pandemic [17]. Barber etal. reported that women in compare with men had higher worry and the more consideration of health behaviours against Covid-19 [18].

From the results, the risk of illness and fatality of women due to COVID-19 was increased by age. The age-dependency of patients was found significant as compared to healthy individuals. The results of the present study on the mortality age distribution as a result of COVID-19 infection (odd ratio = 8.97 in women above 80 years old) was found similar to other studies. For further details, the rate of fatality in Spain, Japan, Italy, and China was the most at the ages above 70 [13, 19, 20]. As per data on average age of female in Ardabil province (as of 2016 census results), it was older compared with that of male in Ardabil province (31.5 vs. 30.9) and Iranian female in general (31.5 vs.31.3) [21]. However, as the years pass, the increase in the gap between the average age of male and women in Iran has been estimated, thus resulting in higher percentage of older women to men. Accordingly, the age-dependent susceptibility to COVID-19 resulted in higher fatality of female of Ardabil province.

The other risk factor of mortality by COVID-19 is to being subjected to non-communicable diseases (NCDs). The individuals underlying NCDs such as cardiovascular disease (CVD), diabetes, hypertension, and chronic respiratory disease are at great risk of death by COVID-19. Therefore, it is vital for people living with NCDs to properly manage their diseases, avoid unhealthy life-style risk factors (e.g., smoking, obesity, and alcohol), and more importantly, obey basic protective measures with respect to COVID-19. Since, the patients with NCDs are more likely to die of the current pandemic, a special care has been devoted to diminish the risk of COVID-19 infection of those suffering from NCDs. From the results of this study, among the patients with NCDs, a large proportion of the study cases suffered from chronic diseases and with CVD accounted for approximately half of the female with COVID-19. Due to somehow lifelong nature of these diseases, the women with chronic and cardiovascular diseases have to approach hospitals and healthcare centres on a regular basis. Hence, they are at higher risks of infection under COVID-19 epidemic. The reports are replete with significantly higher rate of fatality under COVID-19 and NCD synergistic pandemic, syndemic, which is especially of concern for low-income and marginalised individuals who undergo economic and social inequalities and usually do not access proper health services [22]. However, the lack of awareness and some other factors that were taken into account in this study, have roles in susceptibility to infection by COVID-19 and the worst consequences. For instance, more than half of the women hospitalised with CIVID-19 were in contact with unknown resources and one third had travelled outside their residence place, and contacted familiar patients and patients before diagnosis of their illness. This have increased the likelihood of infection and death of the study case group by the time control group strictly followed the cautions and rarely attended dangerous or risky people/places.

On the other hand, the cases with obesity and diabetes devoted 40.6% and 36% of infected female while lung and metabolic diseases accounted for small number of cases. The above-mentioned causes are comparable for all individuals with NCDs and exposed them to higher risks of COVID-19 infection and death. However, obesity is one of the influential factors that puts people at higher risks of infection and fatality by this global pandemic, regardless of their age [23]. The change in immune function of body in obese people has been well-documented [24]. The risk is gradually grown as the body mass index (BMI) increases [23, 25]. In this study, more than 40% of case group were obese, which was significantly higher than control group. In a larger scale, the prevalence of obesity in female of Ardabil province is higher than the average in Iran [26]. Consequently, obesity can be assumed as one of the leading factors of higher rate of female fatality due to COVID-19 in the studied case group. It should be pointed out that the habit of smoking was negligible among the case and control groups and was not accordingly assessed as a potential factor.

Together with the discussed causes, a number of behaviours boosts the susceptibility of infection with COVID-19, if not conducted. Among the studied case and control participants, wearing mask and gloves and implementation of disinfectants in the last 14 days were questioned as of the important healthy behaviours restricting the chance of infection. Practically, a significant difference was found between the case and control groups in wearing mask and gloves. However, slightly more than half of the case group participants remarked the application of disinfectants, which was comparable with control group. Therefore, the faulty implementation of health protocols by case group could be another cause factor of women infected with COVID-19. These protective measures have been extensively emphasised by government authorities and health organisations as primary factors to reduce the spread of infection [27].

### Limitations

There were some limitations to the present study, including the fact that the observance of social distance as one of the key health protocols could not be tested in the present study. Also, the type of mask and disinfectant used by the subjects were not examined in this study.

## Conclusion

In conclusion, the higher rate of the fatality of women in Ardabil province due to COVID-19 could be resulted from the older average age, the prevalence of obesity, and failure in following the protective measures and healthy behaviours. The most important issue regarding the higher rate of the fatality of women in Ardabil province due to COVID-19 can be more health behaviors, including participation in gathering and also having underlying chronic diseases. Some other factors such as incomplete data on individuals affected by COVID-19 across rural areas and far regions, effect of some other demographic and/or socio-economic factors, which put women at higher risks of mortality due to COVID-19.

Future studies should be designed and conducted in order to support the health of women living with NCDs especially during the pandemic. The priority in vaccination should be given to more vulnerable groups including old people and patients with NCDs and obesity. Special emphasis should be given to the increase of women awareness on the importance of healthy diet and life-style. The role of physical activity and maintaining the mental health of women during outbreak should be also taken into account.

## Data Availability

The datasets used and/or analysed during the current study available from the corresponding author on reasonable request.
